# Targeting inflammation or remodeling in asthma? Is there a right way?

**DOI:** 10.3389/fmed.2023.1241920

**Published:** 2023-11-23

**Authors:** Kalliopi Domvri, Konstantinos Porpodis

**Affiliations:** ^1^Laboratory of Histology-Embryology, Medical School, Aristotle University of Thessaloniki, Thessaloniki, Greece; ^2^Laboratory of Pathology, George Papanikolaou Hospital, Thessaloniki, Greece; ^3^Pulmonary Department, Medical School, Aristotle University of Thessaloniki, George Papanikolaou Hospital, Thessaloniki, Greece

**Keywords:** asthma, inflammation, airway remodeling, biologics, modification effect

## Introduction

Underlying pathologies of asthma include inflammation and airway remodeling with structural changes including increased smooth muscle mass, thickening of the airway wall, mucus hypersecretion, angiogenesis and alterations in the extracellular matrix (1). The structural changes affect both large and small airways and can lead to airflow imitation, impaired clearance of inflammatory cells and mucus, and altered responsiveness of the airways to triggers, all of which contribute to the persistence of inflammation (2). A feedback cycle of cytokines and growth factors constantly promotes inflammation and remodeling in the respiratory mucosa (3).

In addition, airway remodeling begins early in the age of 2–4 years old, before clinical diagnosis of asthma and it has been related to all stages of asthma severity (4). According to most animal models, airway remodeling and inflammation are two processes that might occur in parallel and in particular from 1 year to school age whereas from school age and later life, it has been reported that remodeling is stable and inflammation continues (4). However, there is no answer to whether airway remodeling is increasing overtime. What we know is that inflammation and remodeling persist even in those whose symptoms have been resolved.

## Remodeling and inflammation relationship

Little data exist and there is no consensus about the temporal and even causative relationship between airway remodeling and inflammation (5). According to the conventional hypothesis, repeated cycles of acute chronic inflammation lead to airway remodeling, thus, targeting the inflammation is a widely accepted, an evidence-based strategy and an effective approach in the treatment of asthma. It is important to note that while targeting airway inflammation is crucial in asthma management, asthma is a complex and heterogeneous disease. Besides, what if airway remodeling acts as a protective response to limit bronchoconstriction and systemic absorption of inflammatory mediators from the airway lumen? Therefore, a comprehensive approach that considers all aspects of asthma pathophysiology is necessary for effective asthma management.

Furthermore, an alternative challenging point of view of this conventional hypothesis is that inflammation and remodeling might be triggered by the same underlying problem but progress independently (5). Thus, if we hypothesize that chronic inflammation does not lead to airway remodeling, and they act independently, the origin of airway remodeling is not the issue and the need for novel therapies for directing airway remodeling is at need.

As the most prominent feature of airway remodeling is airway muscle mass, bronchial thermoplasty has the role to target it as an irreversible surgical treatment that treats the large airways. A follow-up of three randomized controlled trials has concluded the safety and effectiveness of bronchial thermoplasty after 10 years in persistent asthma (6). However, until more questions are answered, this non-pharmaceutical procedure might be having the greatest application to Th-2 low asthmatics.

## Discussion

Until further alternatives exist to directly target airway remodeling, the question remains: can airway remodeling be reversed? A way to reverse airway remodeling is speculated to occur by targeting airway inflammation. Regarding this research hypothesis, inhaled corticosteroids and other inflammatory treatments have inconsistent results based mostly in *in vivo* models which are not always reliable since most remodeling characteristics are spontaneously reversed (1). Furthermore, limited studies exist on bronchial biopsies regarding the assessment of human airway remodeling after biologic agents' treatment in asthmatic patients. Biologics such as mepolizumab (anti-IL-5), benralizumab (anti-IL-5R) and omalizumab (anti-igE) may have the potential to promote disease modification (6). Interestingly, biologics have shown some effects on the reduction of the characteristics of airway remodeling (7). In specific, the first study to evaluate the effect of a biologic agent on remodeling was by Flood-Page et al. (8), studying the effect of anti-IL-5 treatment in 24 atopic asthmatics after 3 mepolizumab infusions. They concluded that the expression of extracellular matrix proteins was significantly reduced, proposing a mechanism of action due to the reduction of eosinophils producing TGF-β1. In another study, a computational model predicted a significant reduction of airway smooth muscle mass after benralizumab treatment (9). More recently, in a study on 13 severe allergic asthmatics after omalizumab treatment led to modest reductions up to 5% decrease in the percent wall area (10) whereas tezepelumab (anti- thymic stromal lymphopoietin) treatment had no significant effect on basic membrane thickness and epithelial integrity in a double-blind, randomized, placebo-controlled trial (11). Recently, in the European Respiratory Society International Congress 2023, we presented preliminary data of MESILICO study (NCT04612556) showing that one year of mepolizumab treatment resulted in the improvement of airway remodeling in patients with late onset severe eosinophilic asthma and fixed obstruction (12). In specific, in our study, anti-IL-5 treatment reduced basement membrane thickness, smooth muscle area, epithelial damage and tissue eosinophil numbers (12).

Up until now, which features of airway remodeling might be related to certain phenotypes or endotypes is not known and further research is at need (13). Upon this concept, monitoring airway remodeling during clinical trials is crucial for severe clinical phenotypes such as severe asthmatics with fixed airflow obstruction, asthmatics with severe eosinophilia or late onset asthmatics, speculated to be related to airway remodeling driven by chronic inflammation (14, 15). Indeed, “fixed” airflow obstruction, unresponsive to current therapies, even with high-dose systemic glucocorticoids, can be distinguished from a “reversible” one as demonstrated by lung function normalization by the introduction of biological therapies (2).

Thus, targeting airway inflammation appears to impact airway remodeling through the modification effects of biologic agents that target markers of inflammation. Evidence indicates that in addition to targeting eosinophils, IL-5 and anti-IL-5 biologics may have a direct role on airway epithelial cells (16). Toward these directions, registered clinical trials investigate the impact of biological agents on airway remodeling to shed more light. Busse et al. (17) suggest that studies investigating disease-modifying potential of biologics should consider appropriate end-point selection such as change in airway structural abnormalities, appropriate length of trial, age of study population and comorbidities in the patient population. Clinical trials that are currently evaluating the reversing effect of biologics on airway remodeling include mepolizumab (NCT04612556, NCT03797404, NCT05708300), benralizumab (NCT04365205, NCT03953300) and tezepelumab (NCT05651841).^1^

**Figure 1:**
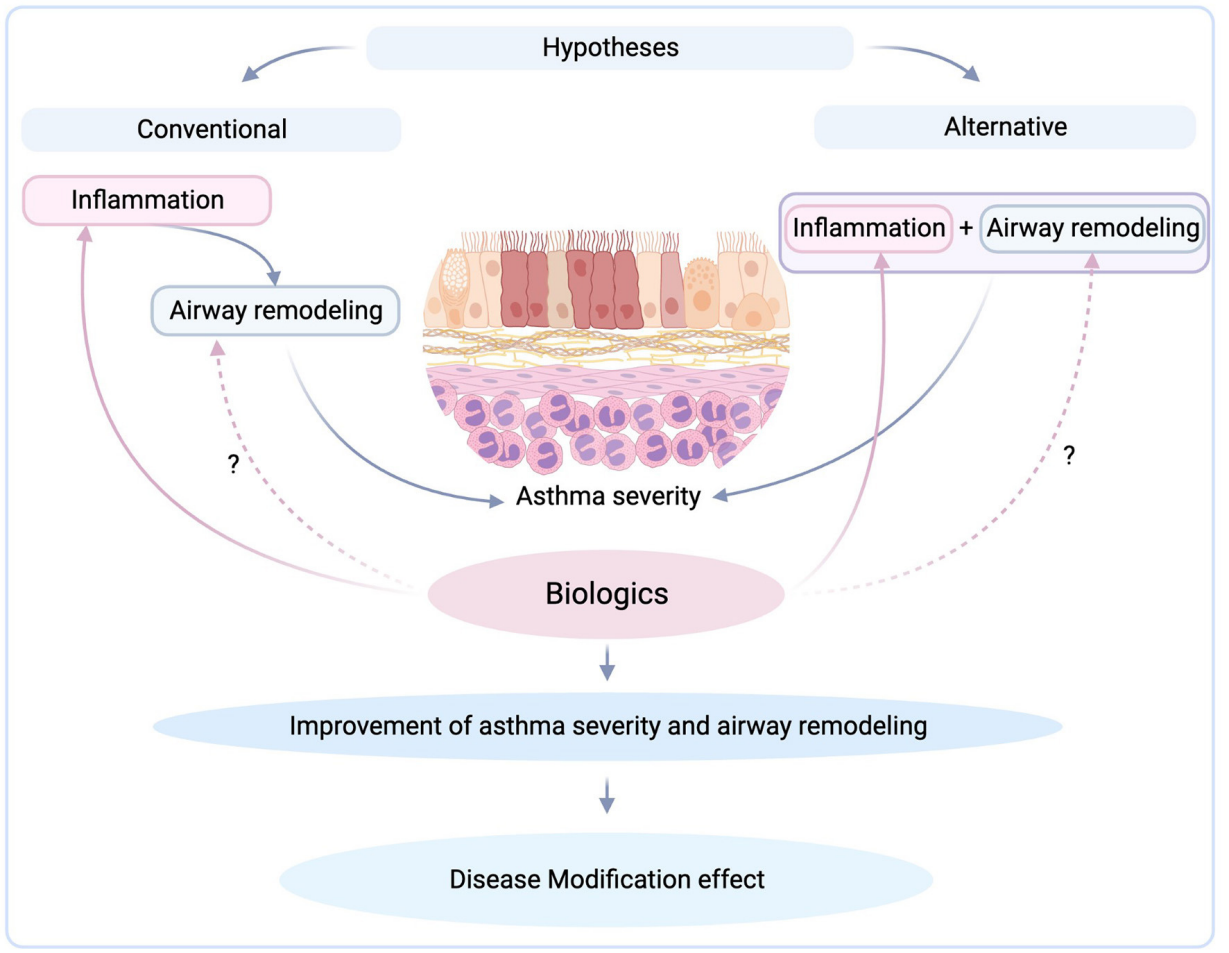
Targeting inflammation by biologics and their possible modification effect (created with BioRender.com).

Overall, is targeting inflammation or remodeling the right way to treat asthma (Figure 1)? Currently, there are not enough data to show us the right way. Until we know more, biologics seem to be the answer in the treatment of severe asthma regarding both inflammation and remodeling. Although limited data exist on the proven effects of biological therapies on airway remodeling in clinical trials, beneficial effects have been reported. Future clinical trials will shed more light by investigating not only the drug effects on inflammation and remodeling but also the evolution of these two processes on a long-term basis.

## Author contributions

KD and KP: conceptualization and writing and editing. All authors contributed to the article and approved the submitted version.

## Footnotes

^1^Available online at: https://clinicaltrials.gov/search?cond=Severe%20Asthma&term=Airway%20Remodeling&aggFilters=status:not%20rec.
